# The impact of genital lichen sclerosus in men and women on quality of life: a prospective cohort study

**DOI:** 10.1097/JW9.0000000000000131

**Published:** 2024-01-18

**Authors:** Sandra Jerkovic Gulin, Linnea Liljeberg, Oliver Seifert

**Affiliations:** a Department of Dermatology and Venereology, Ryhov County Hospital, Sjukhusgatan, Jönköping, Sweden; b Division of Cell Biology, Department of Biomedical and Clinical Sciences, The Faculty of Medicine and Health Sciences Linkoping University, Linköping, Sweden

**Keywords:** genital dermatoses, lichen sclerosus, quality of life

## Abstract

**Background:**

Genital lichen sclerosus (LS) is a chronic inflammatory skin disorder that affects both sexes of all ages. The clinical characteristics include erosions, redness, and white plaques with atrophic skin, with symptoms such as pruritus, pain, dysuria, and dyspareunia.

**Objective:**

This prospective cohort study aimed to assess quality of life (QoL) in men and women with genital LS, both before and after treatment, using the Dermatology Quality of Life Index (DLQI) questionnaire.

**Methods:**

Patients diagnosed with genital LS were enrolled continuously in the study and were asked to complete the DLQI questionnaire before treatment and again after individualized treatment 12 weeks apart.

**Results:**

This study included 136 patients (48 females and 88 males) diagnosed with genital LS, with a median age of 62 years (range 18–86). The results showed a statistically significant decrease (*P* < .001) in DLQI score before treatment (median 6.0 [interquartile range (IQR), 1.0–11.0]) compared to after treatment (median 2.0 [IQR, 0.0–4.0)]. In males and females, the median DLQI scores before treatment were 3.0 (IQR, 0.0–10.0) and 8.0 (IQR, 4.5–11.5), respectively, and after treatment were 1.0 (IQR, 0.0–3.0) and 4.0 (IQR, 0.0–9.0), respectively. Females scored significantly higher (*P* < .001) than males.

**Limitations:**

The study’s limited generalizability stems from a small sample size of 136 patients, potentially restricting the application of findings to a broader population with genital lichen sclerosus. Additionally, the 12-week follow-up period may not adequately capture the long-term effects of interventions on quality of life. Reliance on self-reported data through the DLQI questionnaire introduces the possibility of bias, as participants may not accurately represent their symptoms and quality of life. The absence of a control group hinders the ability to attribute observed changes solely to the treatment, and the lack of detail on specific interventions makes it challenging to assess the effectiveness of individualized treatment approaches. The wide age range among participants (18–86 years) introduces potential confounding variables, as different age groups may respond differently to treatment.

**Conclusion:**

The study findings confirmed that individuals with genital LS experience a small decline in QoL, as observed in both males and females. This study also highlights that effective management of genital LS can significantly improve QoL in both sexes.

**What is known about this subject in regard to women and their families?**

- Genital lichen sclerosus (LS) is a chronic inflammatory skin disorder affecting individuals of all ages and genders.
- Clinical characteristics include erosions, redness, and white plaques with atrophic skin, accompanied by symptoms such as pruritus, pain, dysuria, and dyspareunia.
- Limited previous insights into the impact of genital LS on quality of life (QoL).

**What is new from this article as messages for women and their families?**

- Females with genital LS experienced significantly higher QoL impact compared with males, both before and after treatment.
- Effective management of genital LS substantially improved QoL in women, emphasizing the importance of tailored interventions for this population.
- By shedding light on these aspects, this study contributes to the understanding of the nuanced effects of genital LS on the QoL in both men and women.

## Introduction

Lichen sclerosus (LS) is a chronic inflammatory skin disorder that affects both men and women.^1^ It most commonly occurs in the anogenital region in both sexes, but some patients may have an extragenital presentation.^2,3^ The prevalence of LS is approximately 1.7% in females^4^ and 0.07% in males.^5^ The symptoms commonly experienced by both sexes with LS include pruritus, pain, dysuria, and dyspareunia.^1,6–8^ The clinical features of genital LS include erosion, redness, and white plaques with atrophic skin. In men, LS typically affects the glans penis and prepuce and may lead to anatomic abnormalities such as erectile dysfunction, phimosis, paraphimosis, and urinary retention due to scar tissue formation. Penile LS is a common cause of circumcision.^9^ Vulvar scar tissue formation can lead to anatomical deformation, such as destruction of the labia majora, labia minora, and preputium clitoridis,^8^ and the skin may be lichenified due to long-lasting scratching.^8,10^ There is a potential for malignancy, with vulvar squamous cell carcinoma developing in females^11^ and penile squamous cell carcinoma in males.^9^ A skin biopsy may be necessary in cases of uncertainty regarding LS or suspicion of malignancy.^1,8,12,13^

Multiple factors are considered to play a role in the development of genital LS; however, the exact etiology remains unknown.^1,8^

The British Association of Dermatologists guidelines recommend clobetasol propionate 0.05% ointment as the topical corticosteroids (TCS) of choice.^14^ For penile LS limited to the prepuce, circumcision is often curative, whereas more extensive involvement may require other surgical procedures.^1,9^ Genital LS has been associated with decreased quality of life (QoL) in several studies.^15–17^

Despite its prevalence and impact on individuals’ lives, research on the QoL of patients with genital LS is limited. One of the major domains affected by genital LS is sexual functioning. Sexual dysfunction is a common complaint among female patients with genital LS, significantly impacting their QoL.^17–20^ In a case-control study, it was found that 76% of female patients with genital LS experienced vulvar pain, and they also reported greater sexual dysfunction compared to the control group.^21^Another study reported similar findings, with dyspareunia and sexual dysfunction being prevalent among female patients with genital LS.^17^ However, the literature on male patients with genital LS and their QoL is lacking, with limited studies available on this population.^18^ It is important to recognize the impact of sexual dysfunction and pain on the overall mental health and well-being of patients with genital LS.^22^ A study exploring genital self-image and sexual dysfunction in patients with genital LS revealed that they had significantly lower genital self-image and experienced sexual dysfunction.^23^ These findings suggest that addressing sexual dysfunction and pain is crucial for improving the QoL of individuals with genital LS.

To address the gap in knowledge regarding QoL among both male and female patients with genital LS, this prospective cohort study aims to evaluate QoL, as measured by the Dermatology Life Quality Index (DLQI), before and after individualized treatment. The DLQI is a widely used instrument that assesses the impact of skin conditions on patients’ quality of life. By using this validated measure, this study seeks to provide valuable insights into the QoL of individuals with genital LS and how treatment affects QoL.

## Materials and methods

The study was carried out from March 2022 to September 2022 and included 184 patients (both men and women) with genital LS. The diagnosis of genital LS was based on typical clinical findings. Characteristic features included were: white, shiny patches of skin with a porcelain-like appearance, primarily affecting the genital and anal regions, accompanied by pruritus (itching), burning sensation, skin atrophy, pigmentary changes, fine wrinkling, and, in advanced cases, scarring and adhesions leading to labial or foreskin fusion. Informed consent was obtained at the first visit. All participants were asked to complete the DLQI questionnaire at the first visit (before treatment) and at the second visit (after treatment). All patients received written and oral information about the study purpose, method, risks, and data handling. All patients provided written informed consent to participate in the study before participation.

### Population

The study group of 184 participants was recruited from the Division of Dermatology at the Ryhov County Hospital in Jönköping, Sweden. These patients were also part of a parallel prospective case-control study of the skin microbiome of patients with genital LS. The inclusion and exclusion criteria are listed in Table 1. At the first visit, all patients were asked to complete the DLQI questionnaire, and then again at the second visit, 12 weeks later. Out of the total of 184 patients initially included in the QoL study, 136 patients (comprising 48 women and 88 men) completed both DLQI questionnaires correctly. At the first visit, patients were recommended treatment based on their medical history and clinical findings. Treatment regimens encompassed both topical and systemic therapies. Topical corticosteroids, such as clobetasol cream or ointments, were applied once daily during the first month, every other day during the second month, and twice a week during the third month of treatment. Systemic corticosteroids, specifically prednisolone at a daily dosage of 10 mg, were administered for a minimum of 3 months, with subsequent adjustments based on therapeutic efficacy. Additionally, calcineurin inhibitors, either tacrolimus or pimecrolimus, were applied once daily for both sexes. For men, circumcision, either partial or total, was considered. Complementary treatments included the use of topical antifungals and barrier creams. In instances of severe and recalcitrant genital LS in women, systemic treatment regimens incorporated methotrexate and hydroxychloroquine. The treatment was initiated after the first visit, and in some cases of male LS, circumcision was performed between visits. In total, 136 patients (48 women and 88 men) completed the DLQI before and after 12 weeks of individualized treatment.

**Table 1.** Patient inclusion and exclusion criteria

Inclusion criteria	Exclusion criteria
Age >18 years	Not understanding Swedish or for another reason unable to give consent to participate
Diagnosed genital LS by dermatologist	Pregnancy
	Current cancer diagnosis (except from extra genital BCC and SCC)
	Ongoing cancer treatment
	Previous circumcision in men
	Ongoing anti-inflammatory treatment and/or immunomodulating treatment or having discontinued such treatment within the last 2 weeks
	Ongoing treatment with systemic antibiotics and/or topical antibiotics in the sampling area or having discontinued such treatment within the last 4 weeks
	Ongoing treatment with topical corticosteroids and/or topical calcineurin inhibitors in the sampling area within the last week
	Having used antiseptics or disinfectant in the sampling area 24 h before samples being taken
	Individuals with any genital dermosis other than lichen sclerosus

BCC, basal cell carcinoma; LS, Lichen sclerosus; SCC, squamous cell carcinoma.

### Dermatology Quality of Life Index

The DLQI is a validated tool used to assess QoL in patients with skin disorders.^24,25^ It comprises 10 questions that explore the different dimensions of QoL experienced during the previous week, including symptoms and emotions (questions 1 and 2), daily activities (questions 3 and 4), leisure (questions 5 and 6), work or school (question 7), personal relationships and sexual activity (questions 8 and 9), and treatment (question 10). Thus, the total score ranges from 0 to 30, with higher scores indicating greater impairment of QoL.^25,26^

### Statistics

The data collected in the study were entered into a computerized database, and statistical analyses were conducted using the IBM SPSS software (version 29.0, Mac, SPSS). Descriptive analyses were performed for age and mean total DLQI score before and after treatment. The Kolmogorov–Smirnov test confirmed that the variables were non-normally distributed; thus, the median values were chosen for presentation. To compare the age distribution in women and men, as well as the median DLQI score for each question in women and men both before and after treatment, the nonparametric Mann–Whitney U test was used. The nonparametric Wilcoxon signed-rank test was used to analyze the difference between the total DLQI score at the first and second visits, as well as each question’s DLQI score from the first visit to the second visit, for the total population, men, and women. The significance level was set at *P* < .05. The nonparametric Kruskal–Wallis test was used to compare the total and median DLQI scores for each question before and after treatment in different age groups. Bonferroni correction was performed to adjust for multiple comparisons when 3 or more comparisons were made. Spearman’s rank correlation was computed to assess the relationship between age and total DLQI score before and after treatment.

### Ethical consideration

Ethical approval for this study was issued by the Swedish Ethical Review Authority and obtained from November 15, 2021: dnr. 2021-05590-01.

## Results

### Participants

The study group consisted of 136 patients diagnosed with genital LS, including 48 women and 88 men. Table 2 presents the demographic characteristics of the study groups. The median age of the entire population was 62 years, ranging from 18 to 87 years. The median age was 64 years (range 31–87 years) in women and 59 years (range 18–86 years) in men. The age distribution of women was significantly higher than that of men (z = −2.155; *P* = .031). In addition to genital LS, 1 man and 2 women had extragenital LS, and 1 woman had LS and lichen planus.

**Table 2.** Demographics of the study group (n = 136)

	Total (n = 136)	Men (n = 88)	Women (n = 48)
Median age (range), years	62 (18–87)	59* (18–86)	64* (31–87)
Extragenital LS, number of patients	3	1	2
Lichen planus, number of patients	1	0	1

LS, Lichen sclerosus.

* *P* = .031, Mann–Whitney U-test.

The population was divided into 10 different age groups (0–9 years old; 10–19 years old; 20–29 years old; 30–39 years old; 40–49 years old; 50–59 years old; 60–69 years old; 70–79 years old; 80–89 years old; and 90–100 years old).

### Dermatology Quality of Life Index

#### Total DLQI score

The median total DLQI scores (score interval 0–30) at the first doctor’s visit (DLQI-1) and the median DLQI scores at the second visit 12 weeks apart (DLQI-2) are shown in Table 3. In the population, the DLQI-1 median was 6.0 (interquartile range [IQR], 1.0–11.0) and the DLQI-2 median was 2.0 (IQR, 0.0–4.0). In men, the DLQI-1 median was 3.0 (IQR 0.0–10.0) and the DLQI-2 median was 1.0 (IQR, 0.0–3.0). In women, the DLQI-1 median was 8.0 (IQR, 4.5–11.5) and the DLQI-2 median was 4.0 (IQR, 0.0–9.0).

**Table 3.** Median Dermatology Quality of Life Index score at the 2 visits 12 weeks apart

	Total (n = 136)	Men (n = 88)	Women (n = 48)	Z	*P*
DLQI-1, median (IQR)	6.0 (1.0–11.0)	3.0 (0.0–10.0)	8.0 (4.5–11.5)	−3.822	<.001*
DLQI-2, median (IQR)	2.0 (0.0–4.0)	1.0 (0.0–3.0)	4.0 (0.0–9.0)	−4.722	<.001*
Z	−5.898	−5.011	−3.152		
P	<.001*	<.001*	.002*		

DLQI-1, Dermatology Quality of Life Index score at the first visit, interval 0–30; DLQI-2, Dermatology Quality of Life Index score at the second visit 12 weeks after the first visit, interval 0–30; IQR, interquartile range.

* Statistical significance, *P* < .05. Wilcoxon signed-rank test.

There was a statistically significant decrease in the median DLQI score after treatment (median 2.0 [IQR, 1–5]) compared to before (median 6.0 [IQR, 1–11]), presented in Table 3. There were significant differences in DLQI scores between men and women before treatment (median 3.0 in men and 8.0 in women) and after treatment (median 1.0 in men and 4.0 in women).

#### Each question’s DLQI score

The mean DLQI scores for each question before and after the treatment are shown in Table 4. Questions 1, 2, 8, and 9 had the highest scores before treatment, with mean scores of 1.5, 0.9, 0.7, and 0.9, respectively. After treatment, questions 1, 2, 8, and 9 still had the highest scores, but with lower mean scores of 0.8, 0.5, 0.5, and 0.7, respectively. Statistically significant decreases were found in questions 1 to 7, but not in questions 8 to 10.

**Table 4.** Mean Dermatology Quality of Life Index score before and after treatment in the population as total with *P* values

	DLQI-1, mean (SD)	DLQI-2, mean (SD)	Z	*P*
Q1	1.5 (1.1)	0.8 (0.8)	−6.226	<.001*
Q2	0.9 (1.0)	0.5 (0.8)	−4.859	<.001*
Q3	0.5 (0.8)	0.1 (0.2)	−5.091	<.001*
Q4	0.7 (0.9)	0.3 (0.7)	−4174	<.001*
Q5	0.5 (0.7)	0.2 (0.5)	−3.860	<.001*
Q6	0.4 (0.7)	0.2 (0.5)	−2.949	.003*
Q7	0.4 (0.7)	0.2 (0.5)	−3.639	<.001*
Q8	0.7 (0.9)	0.5 (0.9)	−2.437	.015
Q9	0.9 (1.1)	0.7 (1.0)	−2.307	.021
Q10	0.3 (0.5)	0.3 (0.6)	−0.218	.827

DLQI-1, Dermatology Quality of Life Index score at the first doctor’s visit, interval 0–3; DLQI-2, Dermatology Quality of Life Index score at the second doctor’s visit 12 weeks after the first visit, interval 0–3; SD, standard deviation.

* Statistical significance *P* < .005. Wilcoxon signed-rank test.

Q + number = number of the answered question on the filled in questionnaire.

Each question’s mean DLQI score (score interval 0–3) before and after treatment in both men and women is presented in Table 5. In men, questions 1, 9, 2, and 8 had the highest mean DLQI scores both before and after treatment (1.1, 0.8, 0.7, and 0.6, respectively, before treatment; and 0.6, 0.6, 0.1, and 0.3, respectively, after treatment). In women, questions 1, 2, 9, and 4 had the highest DLQI scores before treatment (2.2, 1.2, 1.2, and 1.1, respectively), and questions 1, 9, 8, 2, and 4 had the highest DLQI scores after treatment (1.3, 1.0, 0.9, 0.8, and 0.8, respectively).

**Table 5.** Mean Dermatology Quality of Life Index before and after treatment in both sexes with *P*-values

	Male				Female			
	DLQI-1, mean (SD)	DLQI-2, mean (SD)	Z	*P*	DLQI-1, mean (SD)	DLQI-2, mean (SD)	Z	*P*
Q1	1.1 (1.1)	0.6 (0.7)	−4.271	<.001*	2.2 (0.9)	1.3 (0.8)	−4.586	<.001*
Q2	0.7 (0.9)	0.3 (0.7)	−3.879	<.001*	1.2 (1.0)	0.8 (0.9)	−2.939	.003*
Q3	0.4 (0.8)	0.1 (0.3)	−4.117	<.001*	0.7 (0.8)	0.3 (0.5)	−2.976	.003*
Q4	0.4 (0.8)	0.1 (0.4)	−3.359	<.001*	1.1 (1.0)	0.8 (1.0)	−2.532	.011
Q5	0.4 (0.7)	0.1 (0.2)	−4.567	<.001*	0.5 (0.7)	0.4 (0.8)	0.695	.487
Q6	0.3 (0.7)	0.1 (0.3)	−3.185	.001*	0.4 (0.8)	0.3 (0.7)	−0.660	.509
Q7	0.3 (0.6)	0.1 (0.3)	−3.421	<.001*	0.6 (0.9)	0.4 (0.7)	−1.710	.087
Q8	0.6 (0.8)	0.3 (0.6)	−2.942	.003*	1.0 (1.1)	0.9 (1.1)	−0.616	.538
Q9	0.8 (1.0)	0.6 (0.9)	−2.061	.039	1.2 (1.2)	1.0 (1.2)	−1.110	.267
Q10	0.3 (0.6)	0.2 (0.5)	−1.895	.058	0.3 (0.5)	0.4 (0.8)	−1.594	.111

DLQI-1, Dermatology Quality of Life Index score at the first doctor’s visit, interval 0–3; DLQI–2, Dermatology Quality of Life Index score at the second doctor’s visit 12 weeks after the first visit, interval 0–3; SD, standard deviation.

* Statistical significance *P <* .005. Wilcoxon signed-rank test.

Q + number = number of the answered question on the filled in questionnaire.

There were statistically significant differences in mean DLQI scores for questions 1, 2, and 4 in men compared with women before treatment, but not in questions 3 or 5–10, as shown in Table 6. There were statistically significant differences in the mean DLQI scores for questions 1–5 and 8 in men compared to women after treatment, but not in questions 6, 7, 9, or 10 (Table 6).

**Table 6.** Comparison of mean Dermatology Quality of Life Index before and after treatment in men compared to women

	Male DLQI-1, mean (SD)	Female DLQI-1, mean (SD)	Z	*P*	Male DLQI-2, mean (SD)	Female DLQI-2, mean (SD)	Z	*P*
Q1	1.1 (1.1)	2.2 (0.9)	−5.512	<.001*	0.6 (0.7)	1.3 (0.8)	−5.489	<.001*
Q2	0.7 (0.9)	1.2 (1,0)	−2.970	.003*	0.3 (0.7)	0.8 (0.9)	2.849	.004*
Q3	0.4 (0.8)	0.7 (0.8)	−2.170	.030	0.1 (0.3)	0.3 (0.5)	−3.270	.001*
Q4	0.4 (0.8)	1.1 (1.0)	−4.246	<.001*	0.1 (0.4)	0.8 (1.0)	−5.085	<.001*
Q5	0.4 (0.7)	0.5 (0.7)	−1.047	.295	0.1 (0.2)	0.4 (0.8)	−4.074	<.001*
Q6	0.3 (0.7)	0.4 (0.8)	−0.012	.990	0.1 (0.3)	0.3 (0.7)	−2.490	.013
Q7	0.3 (0.6)	0.6 (0.9)	−1.323	.186	0.1 (0.3)	0.4 (0.7)	−2.766	.006
Q8	0.6 (0.8)	1.0 (1.1)	−2.182	.029	0.3 (0.6)	0.9 (1.1)	−3.364	<.001*
Q9	0.8 (1.0)	1.2 (1.2)	−1.674	.094	0.6 (0.9)	1.0 (1.2)	−1.939	.053
Q10	0.3 (0.6)	0.3 (0.5)	−0.45	.964	0.2 (0.5)	0.4 (0.8)	−2.080	.038

DLQI-1, Dermatology Quality of Life Index score at the first doctor´s visit, interval 0–3; DLQI-2, Dermatology Quality of Life Index score at the second doctor´s visit 12 weeks after the first visit, interval 0–3; SD, standard deviation.

* Statistical significance *P* < .005. Mann–Whitney U test.

Q + number = number of the answered question on the filled in questionnaire.

#### Age groups’ DLQI score

**Fig. 1.**
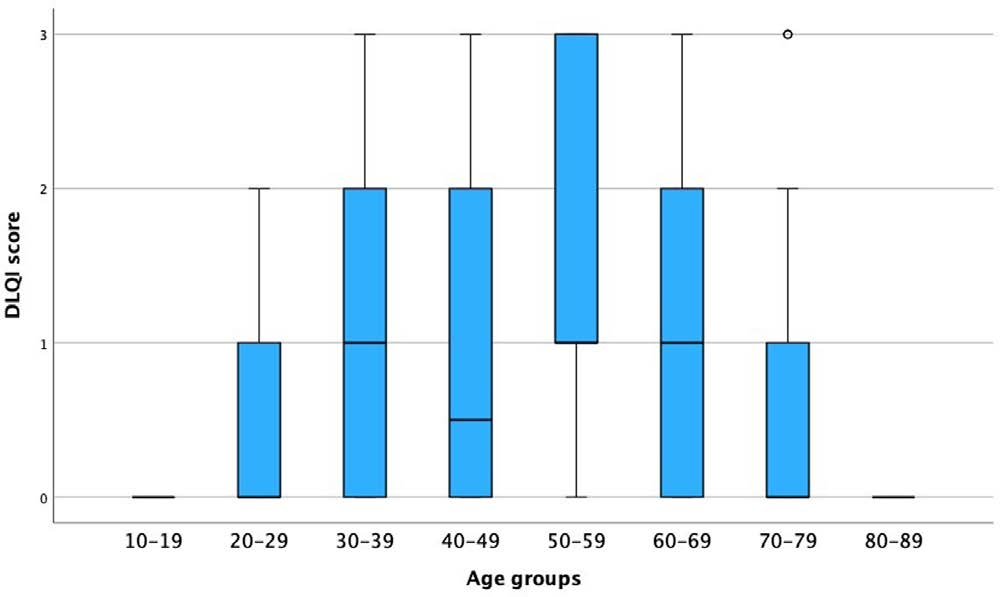
Box plot of DLQI score on question 9 before treatment in different age groups. DLQI score, Dermatology Quality of Life Index score, interval 0–3; — = median; ∥ = interquartile range Q1–Q3; ∣ = range, lower, upper.

A comparison of the different age groups showed a significant difference in the scoring of Question 9, as depicted in Figure 1. Patients aged 50 to 59 showed significantly higher DLQI scores compared to patients aged 70 to 89. There was no significant difference regarding the other questions.

#### Relationship between age and total DLQI score

**Fig. 2.**
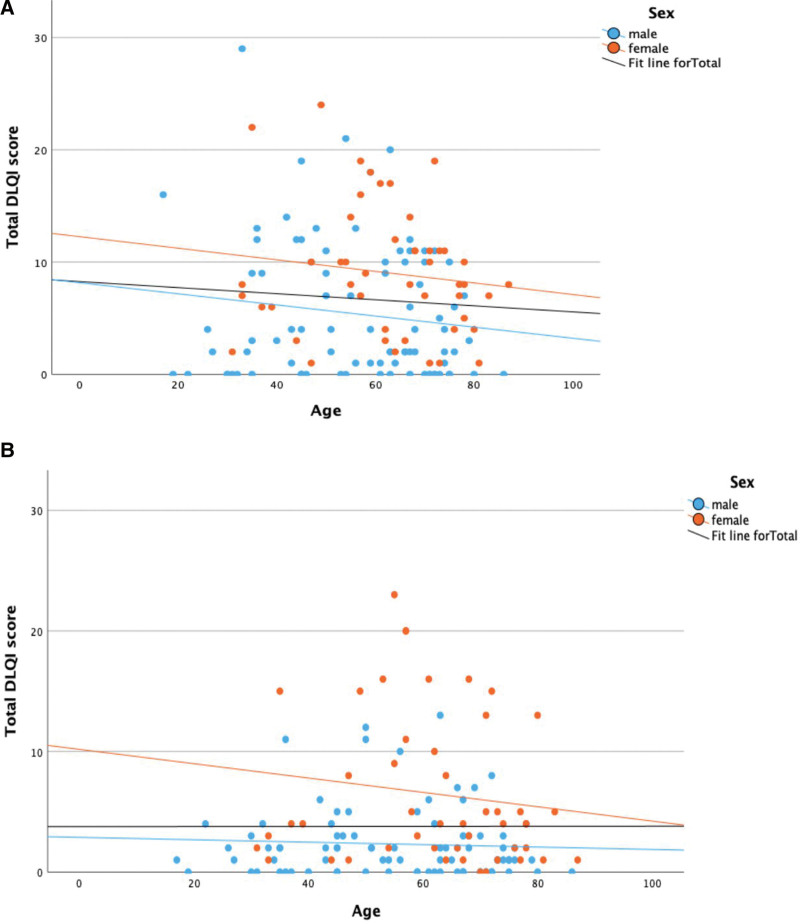
(A) Scatter plot of total DLQI score before treatment dependent on age. (B) Scatter plot of total DLQI score after treatment dependent on age. DLQI score, Dermatology Quality of Life Index score, interval 0–30.

The association between age and total DLQI score, both before and after treatment, is depicted in Figure 2A,B, respectively. Before (r [df = 134] = 0.046; *P* = .594) and after treatment (r [df = 134] = −0.037; *P* = .665), there was no significant linear correlation between age and total DLQI score.

## Discussion

This study aimed to determine QoL among male and female patients with genital LS both before and after treatment, as measured by the DLQI questionnaire. The median total DLQI score before treatment was 6.0, indicating a moderate decrease in the QoL. However, after treatment, the median total DLQI score improved significantly to 2.0, representing a shift from a moderate to a mild effect on QoL. Before treatment, female patients had a higher median total DLQI score of 8.0 compared with male patients (median total DLQI score = 3.0). This comparison revealed a greater decrease in QoL among women, with medians indicating a moderate effect for women and a mild effect for men. In both sexes, treatment resulted in a significant decrease in the DLQI score.

Several studies have reported an impaired QoL in patients with genital LS. Women in particular have been found to experience a moderate effect on QoL in multiple studies, with 1 study reporting a mean DLQI score of 7.7,^17^ while 3 other studies reported median DLQI scores of 6.0^16,27^ and 5.0,^28^ respectively. Another study reported a significant effect on QoL in women with genital LS, with a mean DLQI score of 11.9.^29^

There is little data regarding QoL in male patients with genital LS, as in female patients. A study assessed QoL in male patients with genital LS, measured by the DLQI questionnaire, and reported a significant impairment of QoL with a median DLQI score of 15.0.^30^ Another study in men with genital LS reported a significant impairment of QoL, with a median DLQI score of 17.0.^31^ A prospective study in men with genital LS reported a moderate impairment of QoL, with a median DLQI score before treatment of 7.0.^32^ Overall, these studies suggest that male patients with genital LS have a moderate to significant impairment in QoL, similar to the findings in women. This differs from the results of this study, in which mild impairment in QoL was observed in men before treatment. Possible factors that contribute to this difference are the relatively small sample sizes of these studies and the inclusion of patients. Zucchi et al.^30^ included a similar population in a pilot prospective study on age, diagnosis, and previous treatment. However, the study population comprised only 21 males with genital LS, which may limit the power of the study to draw conclusions. Kyriakou et al.^31^ retrospectively included 41 male patients with genital LS. The males included had biopsy-proven LS accompanied by pruritus.^31^ Casabona et al.^32^ included 45 male patients with genital LS in their prospective study. The males included were those who had failed to experience improvement after a minimum of 6 months of TCS treatment or who requested another treatment other than circumcision and TCS.^32^ Hence, the study population comprised men with penile LS resistant to TCS and, therefore, experienced a more severe symptom burden, which may be reflected in the DLQI scoring.

The lack of studies on QoL among male patients with LS is a notable gap in the literature. Men with genital LS may encounter unique challenges related to the disease, including sexual dysfunction, which may affect their QoL.^8^ This study found a higher total DLQI score before treatment in female patients compared with men, suggesting a more significant impact of genital LS on QoL in women than in men. This may be because women with genital LS experience more severe and frequent symptoms. Future studies should focus specifically on the experiences of male patients with LS to better understand their QoL, treatment needs, and other related factors. By addressing this research gap, healthcare professionals can provide more targeted and effective care for patients with genital LS, regardless of sex.

The decrease in the median DLQI score after treatment compared with before treatment observed in this study was statistically significant, providing evidence of the treatment’s positive impact on the QoL of patients with LS. The effect size (Cohen’s d) indicated that the change in DLQI score was of moderate to large clinical significance, further highlighting the improvement in QoL. These findings are consistent with previous studies that reported impaired QoL among patients with LS and a positive impact of treatment on QoL.^16,27,30,31^

This study’s findings underscore the importance of considering the impact of genital LS on patients’ QoL when determining treatment options. A treatment that provides optimal symptom control but causes significant side effects or necessitates frequent medical consultations may not be the most appropriate choice for patients with low tolerability for such factors.

The highest scores in the questionnaire were observed for questions 1 (symptoms), 2 (emotions), 8 (personal relationships), and 9 (sexual activity). It has been previously reported that sexual activity tends to score the highest on QoL questionnaires for patients with genital LS. In 1 study, sexual dysfunction scored the highest when using the vulvar quality of life index questionnaire in women,^15^ whereas another study found that sexual dysfunction scored the highest when using the DLQI questionnaire.^29^ Sexual dysfunction in patients with genital LS, particularly in women, has been reported in multiple studies.^17,20,21^ Therefore, managing sexual dysfunction and improving QoL are crucial aspects of LS management. Healthcare providers should encourage patients to openly discuss their sexual difficulties to ensure proper management and support, which may lead to improved sexual functioning and, subsequently, improved QoL.

The observed significant decrease in DLQI scores following treatment in this study offers supporting evidence of the validity and responsiveness of the DLQI questionnaire in assessing the impact of LS on patients’ QoL.

It is essential to acknowledge the potential limitations of the DLQI, including its emphasis on skin disease and possible lack of sensitivity to other QoL aspects that may be affected by LS.

The median age of onset of LS is typically in postmenopausal women, with a peak incidence between the ages of 60 and 70 years.^4^ Consistent with this finding, the population in this study had a median age of 62 years. Interestingly, the median age was significantly higher in women than in men, with median ages of 64 and 59 years in women and men, respectively. This is consistent with a previous study that found a higher median age in women with genital LS than that in men.^33^ The age distribution of patients with LS is a critical consideration, as it can have implications for disease management and treatment. For instance, older patients may have comorbidities or other factors that must be considered when creating a treatment plan. Furthermore, older patients may have distinct treatment preferences and goals compared with younger patients.

The observation that age was not a significant predictor of the total DLQI score before or after treatment implies that the influence of LS on QoL does not depend on age. This finding is crucial because it indicates that individuals of all ages may benefit from treatment and support for symptoms related to LS. Nevertheless, it should be noted that this study may have had inadequate statistical power to detect the significant effects of age on QoL, and larger sample sizes in future research may be necessary to validate these findings.

Several study limitations deserve attention. First, the study only assessed the participants’ QoL before and after 12 weeks of treatment. It would be valuable to conduct a long-term follow-up to investigate the sustainability of the treatment effects and to assess the long-term impact on patients’ QoL. Second, the study mentioned that treatment regimens included both topical and systemic therapies but did not provide information on the specific outcomes or comparative effectiveness of different treatments. Further research could compare different treatment options to determine the most effective approach for managing genital LS. The study also did not explore potential risk factors or comorbidities associated with genital LS and its potential effect on DLQI. The comparative effectiveness of different treatment regimens remains underexplored, leaving a void in identifying the optimal approach for managing genital LS. Furthermore, little attention has been given to investigating potential risk factors and comorbidities associated with LS that might influence DLQI. Additionally, different patient subgroups may respond differently to therapies, a question that remains unanswered. The study did not explore potential differences in treatment response or outcomes among specific subgroups of patients. Further research could investigate whether certain therapeutic interventions are more effective for specific patient populations.

The study focused on assessing QoL using a validated questionnaire but did not include in-depth qualitative interviews or assessments of patient perspectives and experiences. Including patient narratives and qualitative data could provide valuable insights into the psychosocial impact, coping strategies, and patient preferences related to genital LS. The study briefly mentioned that questions on the DLQI assessed personal relationships and sexual activity. However, the study did not provide specific details on how sexual health was affected by genital LS, and whether treatment had any impact on sexual functioning or satisfaction. Further research could assess the impact of genital LS on sexual health and explore interventions to address sexual concerns in patients. The implementation of the DLQI questionnaire as a quantitative tool in this study provided an objective measure of the extent to which genital LS affected the QoL of patients before and after treatment. The results of this study reinforce the notion that both men and women with genital LS experience moderate impairment in their QoL. Furthermore, the findings suggest that appropriate management of genital LS can lead to significant improvements in QoL in both male and female patients. Nonetheless, further research is needed to determine the most efficacious and well-tolerated treatments for genital LS.

## Conflicts of interest

None.

## Funding

This study was funded by Futurum—The Academy for Healthcare, Region Jönköping County.

## Study approval

This study was approved by the Swedish Ethical Review Authority on November 15th, 2021 (reference number 2021-05590-01).

## Author contributions

All authors have contributed significantly to this publication. SJG collected the data and contributed to the design of the study and to the writing of the article. LL contributed to the analysis of the results and article writing. OS contributed to the study design, the collection and analysis of the data, and to the writing of the article.

## Patient consent

Informed, written consent was received from all patients included in this study.

## Data availability

All relevant data are within the paper and its supporting information files.

## References

[1] Fergus KB Lee AW Baradaran N. Pathophysiology, clinical manifestations, and treatment of lichen sclerosus: a systematic review. Urology 2020;135:11–9. 31605681 10.1016/j.urology.2019.09.034

[2] Burshtein A Burshtein J Rekhtman S. Extragenital lichen sclerosus: a comprehensive review of clinical features and treatment. Arch Dermatol Res 2023;315:339–46. 36198917 10.1007/s00403-022-02397-1

[3] Virgili A Borghi A Cazzaniga S; GLS Italian Study Group. Gender differences in genital lichen sclerosus: data from a multicenter Italian study on 729 consecutive cases. G Ital Dermatol Venereol 2020;155:155–60. 29368855 10.23736/S0392-0488.17.05819-9

[4] Goldstein AT Marinoff SC Christopher K Srodon M. Prevalence of vulvar lichen sclerosus in a general gynecology practice. J Reprod Med 2005;50:477–80. 16130842

[5] Kizer WS Prarie T Morey AF. Balanitis xerotica obliterans: epidemiologic distribution in an equal access health care system. South Med J 2003;96:9–11. 12602705 10.1097/00007611-200301000-00004

[6] Kwok R Shah TT Minhas S. Recent advances in understanding and managing Lichen Sclerosus. F1000Res 2020;9:F1000 Faculty Rev–369. 10.12688/f1000research.21529.1 PMC7233179 32518626

[7] Lee A Bradford J Fischer G. Long-term management of adult vulvar lichen sclerosus: a prospective cohort study of 507 women. JAMA Dermatol 2015;151:1061–7. 26070005 10.1001/jamadermatol.2015.0643

[8] Gautam MM Singh V Nadkarni NJ Patil SP. Anogenital lichen sclerosus. Indian J Sex Transm Dis AIDS 2020;41:1–9. 33062974 10.4103/ijstd.IJSTD_49_17 PMC7529185

[9] Kwok M Shugg N Siriwardana A. Prevalence and sequelae of penile lichen sclerosus in males presenting for circumcision in regional Australia: a multicentre retrospective cohort study. Transl Androl Urol 2022;11:780–5. 35812204 10.21037/tau-22-29 PMC9262734

[10] Day T Moore S Bohl TG Scurry J. Comorbid vulvar lichen planus and lichen sclerosus. J Low Genit Tract Dis 2017;21:204–8. 28369011 10.1097/LGT.0000000000000307

[11] Bleeker MC Visser PJ Overbeek LI van Beurden M Berkhof J. Lichen sclerosus: incidence and risk of vulvar squamous cell carcinoma. Cancer Epidemiol Biomarkers Prev 2016;25:1224–30. 27257093 10.1158/1055-9965.EPI-16-0019

[12] Krapf JM Mitchell L Holton MA Goldstein AT. Vulvar lichen sclerosus: current perspectives. Int J Womens Health 2020;12:11–20. 32021489 10.2147/IJWH.S191200 PMC6970240

[13] McCarthy S MacEoin N OʼDriscoll M OʼConnor R Heffron C Murphy M. Should we always biopsy in clinically evident lichen sclerosus? J Low Genit Tract Dis 2019;23:182–3. 30688761 10.1097/LGT.0000000000000457

[14] Lewis FM Tatnall FM Velangi SS. British Association of Dermatologists guidelines for the management of lichen sclerosus, 2018. Br J Dermatol 2018;178:839–53. 29313888 10.1111/bjd.16241

[15] Wu M Kherlopian A Wijaya M Fischer G. Quality of life impact and treatment response in vulval disease: comparison of 3 common conditions using the Vulval Quality of Life Index. Australas J Dermatol 2022;63:e320–8. 35932464 10.1111/ajd.13898 PMC9804714

[16] Borghi A Flacco ME Zedde P Toni G Schettini N Corazza M. Does clearance of vulvar lichen sclerosus after a corticosteroid treatment correspond to a decrease in disease-related burden? results from a Cohort Study using pictorial representation of illness and self-measure and the dermatology life quality index. Dermatology 2023;239:81–90. 36382657 10.1159/000526257

[17] Vittrup G Mørup L Heilesen T Jensen D Westmark S Melgaard D. Quality of life and sexuality in women with lichen sclerosus: a cross-sectional study. Clin Exp Dermatol 2022;47:343–50. 34388289 10.1111/ced.14893

[18] Ranum A Pearson DR. The impact of genital lichen sclerosus and lichen planus on quality of life: a review. Int J Womens Dermatol 2022;8:e042. 36000015 10.1097/JW9.0000000000000042 PMC9387966

[19] Pope R Lee MH Myers A. Lichen sclerosus and sexual dysfunction: a systematic review and meta-analysis. J Sex Med 2022;19:1616–24. 36115787 10.1016/j.jsxm.2022.07.011

[20] Kohn JR Connors TM Chan W Liang CS Dao H Jr. Vyas A. Clinical outcomes and adherence to topical corticosteroid therapy in women with vulvar lichen sclerosus: a retrospective cohort study. J Am Acad Dermatol 2020;83:1104–9. 32387654 10.1016/j.jaad.2020.05.006

[21] Haefner HK Aldrich NZ Dalton VK. The impact of vulvar lichen sclerosus on sexual dysfunction. J Womens Health (Larchmt) 2014;23:765–70. 25162790 10.1089/jwh.2014.4805 PMC4158972

[22] Yildiz S Cengiz H Kaya C. Evaluation of genital self-image and sexual dysfunction in women with vulvar lichen planus or lichen sclerosus. J Psychosom Obstet Gynaecol 2022;43:99–106. 33297796 10.1080/0167482X.2020.1857359

[23] Rivera S Flood A Dykstra C Herbenick D DeMaria AL. Genital self-image, sexual function, and quality of life among individuals with vulvar and non-vulvar inflammatory dermatoses. Arch Sex Behav 2022;51:3965–79. 35900677 10.1007/s10508-022-02353-0 PMC9332093

[24] Basra MK Fenech R Gatt RM Salek MS Finlay AY. The Dermatology Life Quality Index 1994-2007: a comprehensive review of validation data and clinical results. Br J Dermatol 2008;159:997–1035. 18795920 10.1111/j.1365-2133.2008.08832.x

[25] Finlay AY Khan GK. Dermatology Life Quality Index (DLQI)--a simple practical measure for routine clinical use. Clin Exp Dermatol 1994;19:210–6. 8033378 10.1111/j.1365-2230.1994.tb01167.x

[26] Hongbo Y Thomas CL Harrison MA Salek MS Finlay AY. Translating the science of quality of life into practice: what do dermatology life quality index scores mean*?* J Invest Dermatol 2005;125:659–64. 16185263 10.1111/j.0022-202X.2005.23621.x

[27] Borghi A Odorici G Scuderi V Valpiani G Morotti C Corazza M. Measuring perceived benefit and disease-related burden in patients affected with vulvar lichen sclerosus after a standard topical corticosteroid treatment Results from a cohort study using Pictorial Representation of Illness and Self-measure and Dermatology Life Quality Index. Dermatol Ther 2020;33:e14334. 32974986 10.1111/dth.14334

[28] Corazza M Virgili A Toni G Valpiani G Morotti C Borghi A. Pictorial Representation of Illness and Self-Measure to assess the perceived burden in patients with chronic inflammatory vulvar diseases: an observational study. J Eur Acad Dermatol Venereol 2020;34:2645–51. 32597539 10.1111/jdv.16637

[29] Van de Nieuwenhof HP Meeuwis KA Nieboer TE Vergeer MC Massuger LF De Hullu JA. The effect of vulvar lichen sclerosus on quality of life and sexual functioning. J Psychosom Obstet Gynaecol 2010;31:279–84. 20701461 10.3109/0167482X.2010.507890

[30] Zucchi A Cai T Cavallini G. Genital lichen sclerosus in male patients: a new treatment with polydeoxyribonucleotide. Urol Int 2016;97:98–103. 26828936 10.1159/000443184

[31] Kyriakou A Patsialas C Patsatsi A Sotiriadis D. Treatment of male genital lichen sclerosus with clobetasol propionate and maintenance with either methylprednisolone aceponate or tacrolimus: a retrospective study. J Dermatolog Treat 2013;24:431–4. 23472631 10.3109/09546634.2013.782385

[32] Casabona F Gambelli I Casabona F Santi P Santori G Baldelli I. Autologous platelet-rich plasma (PRP) in chronic penile lichen sclerosus: the impact on tissue repair and patient quality of life. Int Urol Nephrol 2017;49:573–80. 28161837 10.1007/s11255-017-1523-0

[33] Virgili A Borghi A Cazzaniga S; GLS Italian Study Group. New insights into potential risk factors and associations in genital lichen sclerosus: data from a multicentre Italian study on 729 consecutive cases. J Eur Acad Dermatol Venereol 2017;31:699–704. 27515901 10.1111/jdv.13867

